# Natural Killer (NK) cell responses at female genital mucosa to SIV vaginal challenge

**DOI:** 10.1186/1742-4690-9-S2-P360

**Published:** 2012-09-13

**Authors:** L Shang, J Duan, A Smith, S Wietgrete, M Zupancic, A Haase

**Affiliations:** 1 University of Minnesota, Minneapolis, MN, USA

## Background

Previous studies of SIV transmission at rhesus genital mucosa have identified a vulnerable window of viral replication within the first 3 days post vaginal inoculation. This provides a potential opportunity for host mucosal innate immune responses to control viral infection.

## Methods

In the current work, we examined NK cell (NKG2A+CD3-) responses to SIV vaginal challenge in vaginal and cervical mucosa using IHC and ISH.

## Results

NK cells are maintained at a low baseline level in the genital tissue of naïve animals. In the first week post infection, a small number of NK cells (up to 1500 cells/cm sq) infiltrated into genital mucosa. The initial recruiting signal didn’t require viral replication as shown by equal NK influx in animals challenged with either WT or AT-2 inactivated viruses. The majority of infiltrating NK cells was Granzyme H+ and 40-60% was IFN-gamma+. However, viral inoculation also enhanced the expression of HLA-E in genital tissues, generating an inhibitory environment to NK functionality. In addition, mucosal NK cells appeared to be IL2Rbeta negative, indicating impaired IL-2 and IL-15 signaling. Therefore, the initial influx of NK cells was not able to eliminate early viral infection at genital mucosa. Surprisingly, in the second week, the number of mucosal NK cells rapidly decreased, which is inversely associated with the number of infected cells in genital tissues and is consistent with an increase in the level of sera IP10 and IL18. Interestingly, at the end of the fourth week (the VL set point), the number of NK cells in genital mucosa was partially recovered, probably because of less viral replication due to CD4 depletion and a reduction of sera IP10 and IL18.

## Conclusion

In this study, we found that densities of cervical NK cells are inversely correlated with those of infected cells in animals at Day 5-10 post infection (n=7, p=0.0187).

